# The Role of Biodegradable Magnesium and Its Alloys in Anterior Cruciate Ligament Reconstruction: A Systematic Review and Meta-Analysis Based on Animal Studies

**DOI:** 10.3389/fbioe.2021.789498

**Published:** 2021-11-16

**Authors:** Zhizhong Shang, Dongliang Li, Jinlei Chen, Mingchuan Wang, Baolin Zhang, Xin Wang, Bin Ma

**Affiliations:** ^1^ The First Clinical Medical School of Lanzhou University, Lanzhou, China; ^2^ Department of Spine, Changzheng Hospital, Naval Medical University, Shanghai, China; ^3^ Evidence Based Medicine Center, School of Basic Medical Sciences, Lanzhou University, Lanzhou, China

**Keywords:** magnesium and its alloys, titanium and its alloys, anterior cruciate ligament reconstruction, animal study, systematic review

## Abstract

**Objective:** The actual efficacy of magnesium and its alloy in anterior cruciate ligament reconstruction (ACLR) was systematically evaluated to reduce the risk of translation from animal experiments to the clinic.

**Methods:** Databases of PubMed, Ovid-Embase, Web of Science, CNKI, Wanfang, VIP, and CBM were searched for literature in July 2021. Screening of search results, data extraction, and literature quality evaluation were undertaken independently by two reviewers.

**Results and discussion:** Seven articles were selected for the meta-analysis. The results showed that the mechanical properties of the femoral-tendon graft–tibia complex fixed with magnesium and its alloys were comparable to those fixed with titanium and its alloys, and magnesium and its alloys were superior to titanium and its alloys in promoting new bone formation. In addition, the unique biodegradability made magnesium and its alloys an orthopedic implant with significant therapeutic potential. However, whether the degradation rate of magnesium and its alloy can match the rate of bone-tendon integration, and whether the bioconjugation of bone-tendon after degradation can meet the exercise load still needs to be explored in further detail. Simultaneously, it is necessary for future research to improve and standardize experimental design, result measurement, etc., so as to minimize the risk of transforming animal experimental results into clinical practice.

## Introduction

Anterior cruciate ligament (ACL) injury is one of the most common injuries of the knee, accounting for 20.3% of total knee injury disease (Majewski et al., 2006). The incidence of ACL injury ranges from 32 to 77.4 per million people, with a higher incidence among professional athletes (e.g., soccer, rugby and team handball) (Moses et al., 2012; Zbrojkiewicz et al., 2018). ACL injury has an estimated incidence of over four million new cases annually worldwide, and the population is increasingly younger with the popularity of sport among children and adolescents (Mather et al., 2013).

The most devastating consequence of ACL injury is that osteoarthritis is prone to occur due to instability of the knee joint, which causes physical and mental suffering as well as financial burden to the patients (Mather et al., 2013; Ajuied et al., 2014). Initially, repair is the mainstream treatment of ACL injury; however, multiple randomized controlled trials have demonstrated that patients receiving anterior cruciate ligament reconstruction (ACLR) had better functions of the knee than those receiving ACL repair (Engebretsen et al., 1990; Andersson et al., 1991) and that ACLR could effectively slow the progression of osteoarthritis (Mihelic et al., 2011; Mather et al., 2013). Therefore, ACLR is the current gold standard for treatment of ACL injury and has become an option for nearly half of patients (Mahapatra et al., 2018). However, the clinical failure rate after ACLR is high, up to 25% (Kamath et al., 2011), and the revision rate increases on average by 5.6% per year (Zbrojkiewicz et al., 2018). Especially for athletes, ACL injury is not only devastating to their athletic career (Mahapatra et al., 2018), but only one-half and two-thirds of patients can return to pre-injury levels (Ardern et al., 2011; Ardern et al., 2014).

The main cause of failure after ACLR is the graft spondylolisthesis caused by poor healing of the bone-tendon junction, which leads to relaxation of the knee joint (Chen et al., 2004; Fankhauser et al., 2004). Thus, good graft fixation is critical to ACLR. Currently, screw screws made from various biomaterials such as metal, polymers, and composites are widely used for the fixation of ACLR because of their excellent mechanical properties and appropriate biocompatibility (Fineberg et al., 2000; Lee et al., 2016). However, metal screws (mainly titanium and stainless steel) have many problems in clinical application, for example, the bone loss caused by stress shielding, coupled with hindrance of the recovery of *in situ* tissue, which requires a second operation to remove the fixation device; damage to tendon or ligament graft during implantation; inflammation as a foreign body; and diagnostic image distortion (Chen and Thouas, 2015; Esmaeilzadeh and Setayesh, 2020). Although screws made from bioabsorbable materials overcome defects in metal screws, they may fail during implantation due to their poor mechanical strength. Furthermore, the degradation of screw may cause adverse events such as synovitis, granuloma, and enlargement of the bone tunnel, which limits its usage in clinic (Baums et al., 2006). Therefore, maintaining the mechanical properties and biocompatibility of the fixed material and promoting the integration of the bone-tendon graft in ACLR remains a great challenge.

Currently, biodegradable mentals represented by magnesium (Mg) and its alloys have received extensive attention for their application in artificial implants because they exhibit such advantages as good biocompatibility, degradability, and the elastic modulus being similar to the human bone (Chen, 2017; Han et al., 2019). Not only do these metals have excellent mechanical properties, which can provide fixed strength in the initial stage of bone-tendon healing, but also the implants will gradually degrade in a physiological environment, avoiding the damage caused by secondary removal and bone decline caused by stress occlusion of traditional metal instruments (Yazdimamaghani et al., 2017). Previous experimental studies have also shown that angiogenesis and new bone formation were the key factors affecting bone-tendon interface healing. Magnesium ions could stimulate osteogenic differentiation of stem cells and promote bone tunnel healing and tendon graft binding to the surrounding bone tissue (Li and Zheng, 2013; Cheng et al., 2016a). At present, magnesium and its alloys have shown great therapeutic potential in tendon graft fixation of ACLR; for example, the study by Wang et al. (2018) showed that magnesium alloy could also promote the formation of new bone around the bone tunnel while providing sufficient mechanical strength. The study by Cheng et al. (2016b) showed that magnesium-based screws could increase the expression of BMP-2 and VEGF at the bone-tendon interface, thus promoting the biological binding of the tendon graft to the surrounding bone tissue. However, magnesium and its alloys also have some problems in ACLR. For example, magnesium has high corrosion sensitivity and non-uniform corrosion behavior in the presence of stress and simulated physiological environment rich in chloride ions. The mechanical properties may be reduced significantly after implantation, and the risk of losing fixation functions may increase significantly before complete healing of the bone-tendon (Jafari et al., 2015). Meanwhile, excessive ions and excessive gas due to rapid degradation may cause excessive accumulation of metal ions in local tissues, metabolic overload, and formation of a gas cavity around the implant (Li et al., 2020). In addition, other studies also showed that (Liu et al., 2010) lack of torque of the magnesium-based screw was one of the main causes for the fracture of screw head easily during ACLR.

Therefore, a systematic review was used for the first time in our study to conduct a comprehensive analysis of published animal studies with magnesium and its alloys at home and abroad, to explore the real efficacy and potential risks and its feasibility of clinical transformation in ACLR, so as to reduce the risk of preclinical results to clinical transformation.

## Materials and methods

### Inclusion and exclusion criteria

#### Subjects

Animal models of ACLR were included, with no restrictions on species and modeling method.

#### Interventions

Magnesium and its alloys.

#### Control

Titanium and its alloys.

#### Outcome

1) Ultimate load to failure: the femur-tendon graft–tibia complexes (FTGTC) were stretched by a biomechanical experimental machine to obtain ultimate load to failure.
2) Outcome indicators related to new bone formation
① Trabecular number (Tb. N): 3D reconstruction of bone tissue by Micro-CT and calculation of trabecular number by image analysis software such as INVEON or VGStudio software.
② Trabecular thickness (Tb. Th): 3D reconstruction of bone tissue by Micro-CT and calculation of trabecular thickness by image analysis software such as INVEON or VGStudio software.
③ Trabecular separation (Tb. Sp): 3D reconstruction of bone tissue by Micro-CT and calculation of trabecular separation by image analysis software such as INVEON or VGStudio software.
④ BV/TV: 3D reconstruction of bone tissue by Micro-CT and calculation of BV/TV by image analysis software such as INVEON or VGStudio software.
⑤ Mineral apposition rate: the amount of minerals was obtained by high-resolution-peripheral quantitative computed tomography (HR-Pqct) scan to calculate the mineral apposition rate.
⑥ The width of osteoid: representative Goldner’s Trichrome-stained sections for the width determination of osteoid.
3) Degradation of implants: fracture or corrosion of implants was observed by imaging and residual volume of implants was measured by Micro-CT.

Given the differences in baseline characteristics such as age (between April and October), body weight (between 2.5 and 4.0 kg), and bone tunnel diameter (between 2.1 and 4.0 mm), the healing time of bone and tendon must be different. To facilitate the combinatorial analysis of the outcome measures, we divided the whole follow-up process of the included studies into three measurement periods, which were T1, the initial period (0<T1≤1/3T), T2, the mid-term period (1/3T<T2≤2/3T), and T3, the late-term period (2/3T<T3≤3/3T), with “T” representing the whole follow-up time.

#### Type of study

Control studies were included, with no restrictions on allocation concealment or blind method.

### Search strategy

Computer retrieval was conducted in PubMed, Ovid-Embase, Web of Science, CNKI, Wanfang, VIP, and CBM, and the retrieval deadline was July 2021. The retrieval words were anterior cruciate ligament reconstruction OR anterior cruciate ligament OR ACL OR ACLR AND (magnesium OR Mg OR magnesium alloy OR degradable metal. See Appendix 1 for the full search strategies of each database.

### Literature screening and data extraction

Two trained researchers selected the papers and extracted the data in strict accordance with the inclusion/exclusion criteria, and cross-checked them. In case of disagreement, a third party would decide. Data were extracted according to the preestablished full-text data extraction checklist, including ① basic information: author, year, type of study, baseline characteristics of animals, sample size, type of graft, diameter of bone tunnel, shape of implant, and length of follow-up and ② outcomes: ultimate load to failure, outcome indicators related to new bone formation, and degradation of implants.

### Risk of bias assessment

Based on SYRCLE’s risk-of-bias tool for animal studies (Hooijmans et al., 2014), two trained researchers independently evaluated and cross-checked the inherent risk of bias in the included studies, covering selection bias, implementation bias, measurement bias, follow-up bias, report bias, and other bias from a list of 10 questions or tools. A difference in opinions was negotiated or decided by a third party. The answer to the assessment questions (tools) should be either “yes” which indicated low risk of bias or “no” which indicated high risk of bias. For unclear items, an answer with “unclear” was assigned.

### Quality assessment of evidence

Whether the results of the systematic review of animal studies can lead to clinical translation depends on the quality of the evidence. Based on the GRADE evidence grading system (Guyatt et al., 2008), quantitative indicators were used to assess the evidence quality in the following five aspects: 1) research limitation; 2) inconsistency of results; 3) indirectness; 4) inaccuracy; and 5) publication bias. First of all, the evidence quality of each result was evaluated, and then the evaluation results of each part were integrated to achieve the grades of evidence: high, medium, low, and extremely low.

### Statistical analysis

STATA 16 software was used for statistical analysis. Weighted mean difference (WMD) was regarded as the effect analysis statistic for measurement data, and risk ratio (RR) was used as the effect analysis statistic for dichotomous variables. Both of them used the 95% CI as the effect amount. Heterogeneity of results between studies was assessed by a χ2 test (the significant level for heterogeneity test was *p* = 0.1). Also, *I* ^*2*^fn2 was used to judge the degree of heterogeneity. If the research results were not statistically different, the fixed-effect model would be used for meta-analysis. Conversely, if there was statistical heterogeneity, the source of heterogeneity would be further analyzed, and the random-effect model would be used for meta-analysis after the effect of the obvious clinical heterogeneity was excluded.

## Results

### Literature search results

**FIGURE 1:**
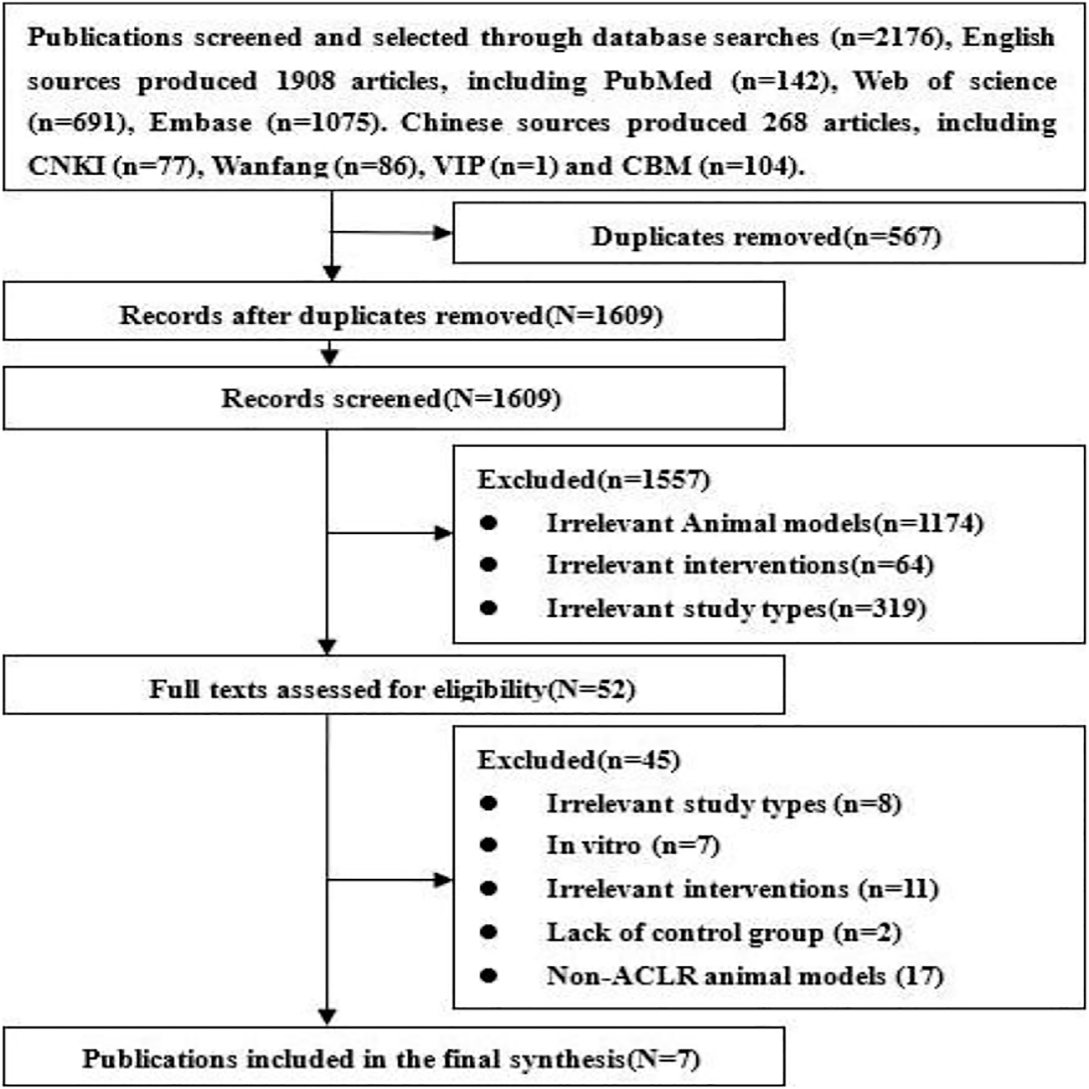
Flowchart of the literature-screening process.

A total of 2,176 references (1,908 English and 268 Chinese publications) were searched through databases. After excluding duplicates and those not meeting the inclusion criteria, seven papers (Cheng et al., 2016b; Cheng et al., 2016c; Diekmann et al., 2016; Wang et al., 2017a; Wang et al., 2017b; Mao et al., 2021; Sun et al., 2021) about graft during ACL reconstruction with magnesium and its alloys were included in our systematic review, and all were in English literature. The flowchart of the literature screening is shown in Figure 1.

### Basic information of studies

Of the seven animal studies included, only one study (Diekmann et al., 2016) was a randomized controlled trial, and the other six studies (Cheng et al., 2016b; Cheng et al., 2016c; Wang et al., 2017a; Wang et al., 2017b; Mao et al., 2021; Sun et al., 2021) were all controlled trials. All experimental animals were New Zealand white rabbits. Animals in six of studies (Cheng et al., 2016b; Cheng et al., 2016c; Wang et al., 2017a; Wang et al., 2017b; Mao et al., 2021; Sun et al., 2021) were male, and one study (Diekmann et al., 2016) did not report the sex of the animals. The body weights of animals were between 2.5 kg (Sun et al., 2021) and 4.0 kg (Mao et al., 2021). Age were between 4 months (Sun et al., 2021) and 10 months (Mao et al., 2021). Samples size were between 36 (Diekmann et al., 2016) and 112 (Wang et al., 2017a). Sources of the reconstructed ACL included semitendinosus (Cheng et al., 2016b; Cheng et al., 2016c), long digital extensor tendon (Diekmann et al., 2016; Wang et al., 2017a; Wang et al., 2017b; Sun et al., 2021), and achilles tendon (Mao et al., 2021). The diameter of the bone tunnel was between 2.1 mm (Cheng et al., 2016b; Cheng et al., 2016c) and 4.0 (Mao et al., 2021) mm. Shapes of all implants were screws. The components of screws in the experimental group included high-purity magnesium (HP Mg) (Cheng et al., 2016b; Cheng et al., 2016c; Wang et al., 2017a; Wang et al., 2017b; Sun et al., 2021) and magnesium alloy (Diekmann et al., 2016; Sun et al., 2021), and the control group included titanium (Cheng et al., 2016b; Cheng et al., 2016c; Wang et al., 2017a; Wang et al., 2017b; Mao et al., 2021) and alloys (Diekmann et al., 2016; Sun et al., 2021). The lengths of screws were between 8 mm (Wang et al., 2017a; Wang et al., 2017b) and 12 mm (Cheng et al., 2016b; Cheng et al., 2016c). The follow-up was between 9 weeks (Cheng et al., 2016c) and 24 weeks (Diekmann et al., 2016). Evaluation criteria for outcome measures included biomechanical testing using the Zwick material testing machine (Cheng et al., 2016b; Cheng et al., 2016c), uniaxial mechanical testing machine (Wang et al., 2017a), and biomechanical testing machine (Mao et al., 2021; Sun et al., 2021). A quantitative assessment of new bone formation was performed by high-resolution-peripheral quantitative computed tomography (Wang et al., 2017b) and Micro-CT (Mao et al., 2021; Sun et al., 2021). The remaining volume of the implants was quantitatively assessed by Micro-CT (Cheng et al., 2016b; Cheng et al., 2016c) and μCT-scans (Diekmann et al., 2016). Basic information of the study subjects was summarized, as shown in Table 1.

**TABLE 1:** Basic information for inclusion in the study.

Author (year)	Country	Study type	Species	Gender	Weight	Age	Sample	Graft	Follow-up
Cheng (2016a)	China	Control	New Zealand white rabbits	Male	—	Skeletal maturation	60	Semitendinosus	12 weeks
Cheng (2016b)	China	Control	New Zealand white rabbits	Male	—	Skeletal maturation	60	Semitendinosus	9 weeks
Diekmann (2016)	Germany	RCT	New Zealand white rabbits	—	3.8 ± 0.2 kg	6 months	36	Long digital extensor tendon	24 weeks
Sun (2020)	China	Control	New Zealand white rabbits	Male	2.5–3.0 kg	4–6 months	60	Long digital extensor tendon	16 weeks
Wang (2017a)	China	Control	New Zealand white rabbits	Male	—	6 months	112	Long digital extensor tendon	16 weeks
Wang (2017b)	China	Control	New Zealand white rabbits	Male	—	Skeletal maturation	64	Long digital extensor tendon	16 weeks
Mao (2021)	China	Control	New Zealand white rabbits	Male	3.5–4.0 kg	9–10 months	39	Achilles tendon	12 weeks

Author + year	Diameter of bone tunnel (mm)	Shape of implants	Interventions		Components of implants		Implant specifications
			Experimental group	Control group	Experimental group (wt%)	Control group (wt%)	
Cheng (2016a)	2.1	Screw	HP Mg	Ti	The HP Mg (99.98)	Titanium (99.8)	Length: 12 mm, core diameter: 2.1 mm, major diameter: 2.7 mm
Cheng (2016b)	2.1	Screw	HP Mg	Ti	The HP Mg (99.98)	Titanium (99.8)	Length: 12 mm, core diameter: 2.1 mm, major diameter: 2.7 mm
Diekmann (2016)	2.7	Screw	MgYREZr alloy	Ti6Al4V	—	—	Length:10 mm, external diameter: 2.6 mm, thread pitch: 0.8 mm
Sun (2020)	2.5	Screw	Mg alloy	Ti6Al4V	Zn-0.81Mn-0.45 Mg	—	—
Wang (2017a)	2.5	Screw	HP Mg	Ti	Mg (99.99)	—	Length: 8 mm, diameter: 3 mm
Wang (2017b)	2.5	Screw	HP Mg	Ti	The HP Mg (99.98)	Titanium (99.8)	Length: 8 mm, outer diameter: 3 mm
Mao (2021)	4.0	Screw	HP Mg	Ti	The HP Mg (99.97)	Titanium (99.53)	—

### Results from assessing the risk of bias and quality of evidence

**FIGURE 2:**
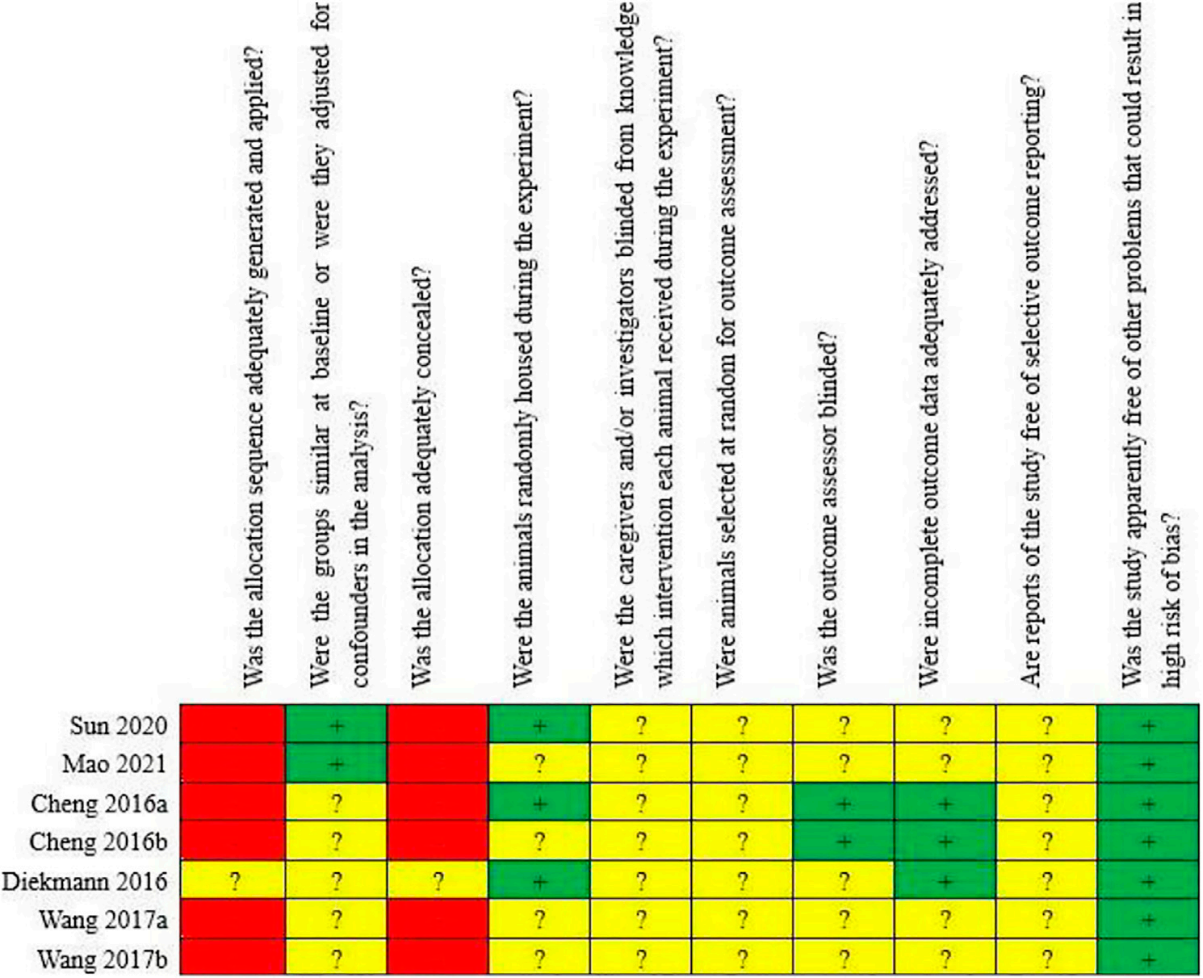
Risk of bias of the individual included animal studies. The items were scored with “yes,” “no,” “unsure.”

**FIGURE 3:**
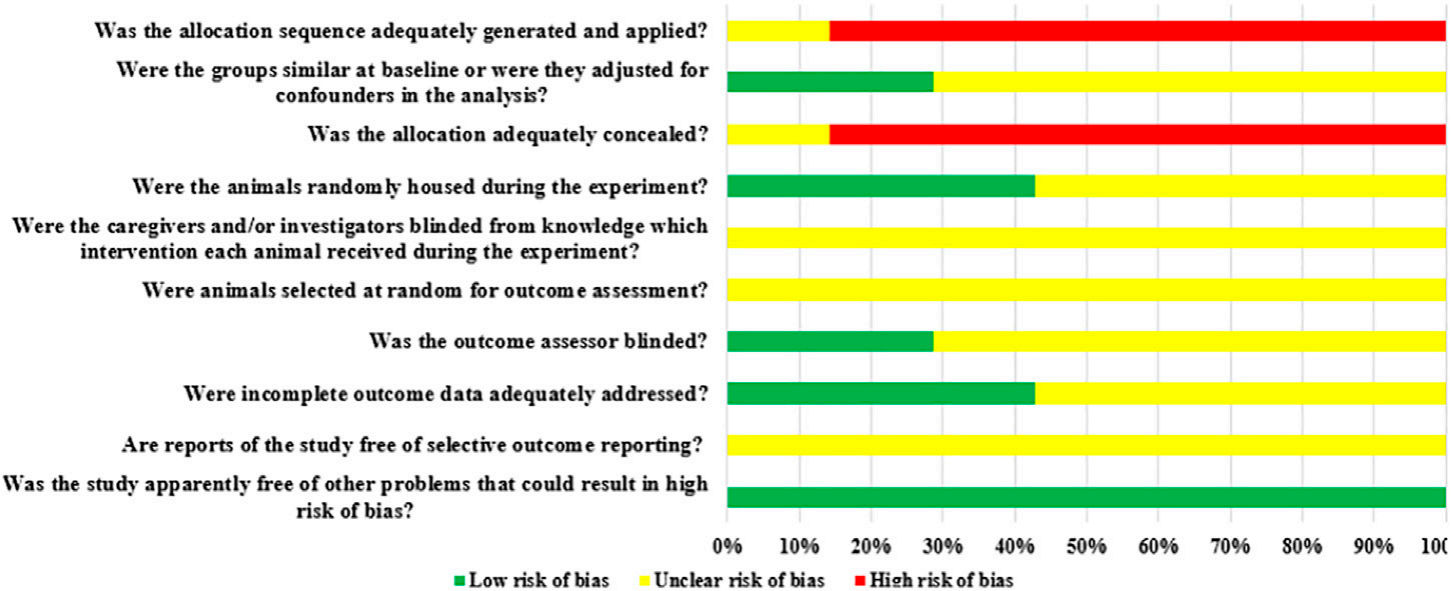
Risk of bias of each item of the SYRCLE tool for overall included studies. Each risk-of-bias item presented as percentages across all included studies, which indicated the proportion of different level risk of bias for each item.

Among the seven included animal studies, only one randomized controlled trial was included (Diekmann et al., 2016) but did not explain the specific random grouping and whether covert grouping was implemented. Only two studies reported the equilibrium of baseline characteristics such as age, sex, and body weight of animals (Mao et al., 2021; Sun et al., 2021). Only three studies reported randomized placement of animals during the experiment (Cheng et al., 2016b; Diekmann et al., 2016; Sun et al., 2021). Based on limited information provided by the included studies, all studies were unable to determine whether or not to blind animal breeders and/or researchers. None of the entire studies reported methods for animal selection during the outcome evaluation. Only two studies reported blinding of outcome assessors (Cheng et al., 2016b; Cheng et al., 2016c). All experimental animals of three studies were included in the final analysis (Cheng et al., 2016b; Cheng et al., 2016c; Diekmann et al., 2016). Although no research protocol was available for any of the studies, all expected results were clearly reported. The risk-of-bias assessment for all studies is shown in Figures 2, 3.

The results from assessing the quality of evidence showed “low” or “very low” quality in the eight outcome measures. The reasons for the degradation of evidence quality included the lack of intrinsic authenticity of original research, the inconsistency of research results, and the possibility of large publication bias. The results of the evidence quality assessment are detailed in Table 2.

**TABLE 2:** Quality of evidence in included systematic reviews with GRADE.

Outcomes	Number of literatures	Research limitation	Inconsistency	Indirectness	Inaccuracy	Publication bias	Quality of evidence
Ultimate load to failure	6 Cheng et al. (2016b); Cheng et al. (2016c); Wang et al. (2017a); Wang et al. (2018); Mao et al. (2021); Sun et al. (2021)	Serious^a^	Serious^b^	Not serious	Serious^c^	Serious^d^	Very low
Tb. N	3 Wang et al. (2018); Mao et al. (2021); Sun et al. (2021)	Serious^a^	Serious^b^	Not serious	Not serious	Serious^d^	Low
Tb. Th	3 Wang et al. (2018); Mao et al. (2021); Sun et al. (2021)	Serious^a^	Serious^b^	Not serious	Not serious	Serious^d^	Low
Tb. Sp	3 Wang et al. (2018); Mao et al. (2021); Sun et al. (2021)	Serious^a^	Serious^b^	Not serious	Not serious	Serious^d^	Low
BV/TV	3 Wang et al. (2017a); Mao et al. (2021); Sun et al. (2021)	Serious^a^	Serious^b^	Not serious	Not serious	Serious^d^	Low
Mineral apposition rate	1 Wang et al. (2017a)	Serious^a^	Serious^b^	Not serious	Serious^c^	Serious^d^	Very low
The width of osteoid	1 Wang et al. (2017b)	Serious^a^	Serious^b^	Not serious	Serious^c^	Serious^d^	Very low
Degradation of implants	5 Cheng et al. (2016b); Cheng et al. (2016c); Diekmann et al. (2016); Wang et al. (2017b); Wang et al. (2018)	Serious^a^	Serious^b^	Not serious	Serious^c^	Serious^d^	Very low

a The design of the experiment with a large bias in random, distributive hiding or blind.

b The confidence interval overlaps less, and I^2^ is larger.

c The confidence interval is too wide or contains invalid values.

d Fewer studies are included and there may be greater publication bias.

### Meta-analysis results

#### Ultimate load to failure

**FIGURE 4:**
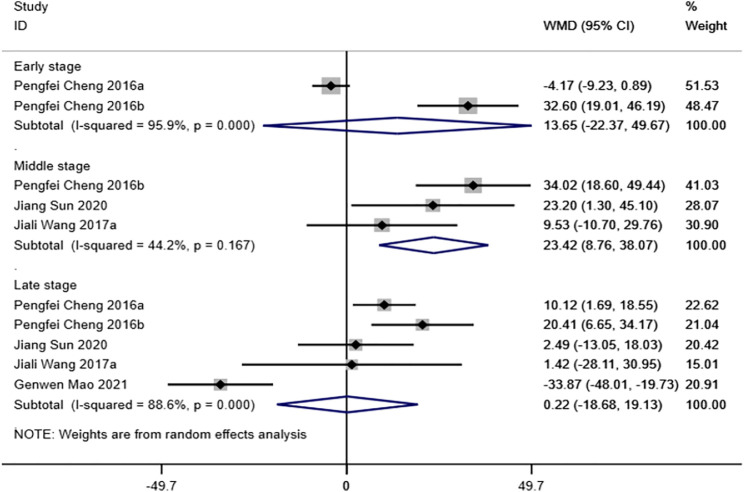
Meta-analysis results of ultimate load to failure of magnesium and its alloys vs. titanium and its alloys.

Five studies (Cheng et al., 2016b; Cheng et al., 2016c; Wang et al., 2017a; Mao et al., 2021; Sun et al., 2021) reported ultimate load to failure of the FTGTC. The results of the random-effect model showed that no statistically significant differences were observed between magnesium and its alloy group and titanium and its alloy group in early and late stages (early stage: WMD = 13.65 [-22.37, 49.67]; late stage: WMD = 0.22 [-18.68, 19.13]). In the middle stage, however, ultimate load to failure of magnesium and its alloy group was significantly higher than that of titanium and its alloy group, and the difference was statistically significant (WMD = 23.42 [8.76, 38.07]). See Figure 4 for further details.

#### Outcome indicators related to new bone formation

**FIGURE 5:**
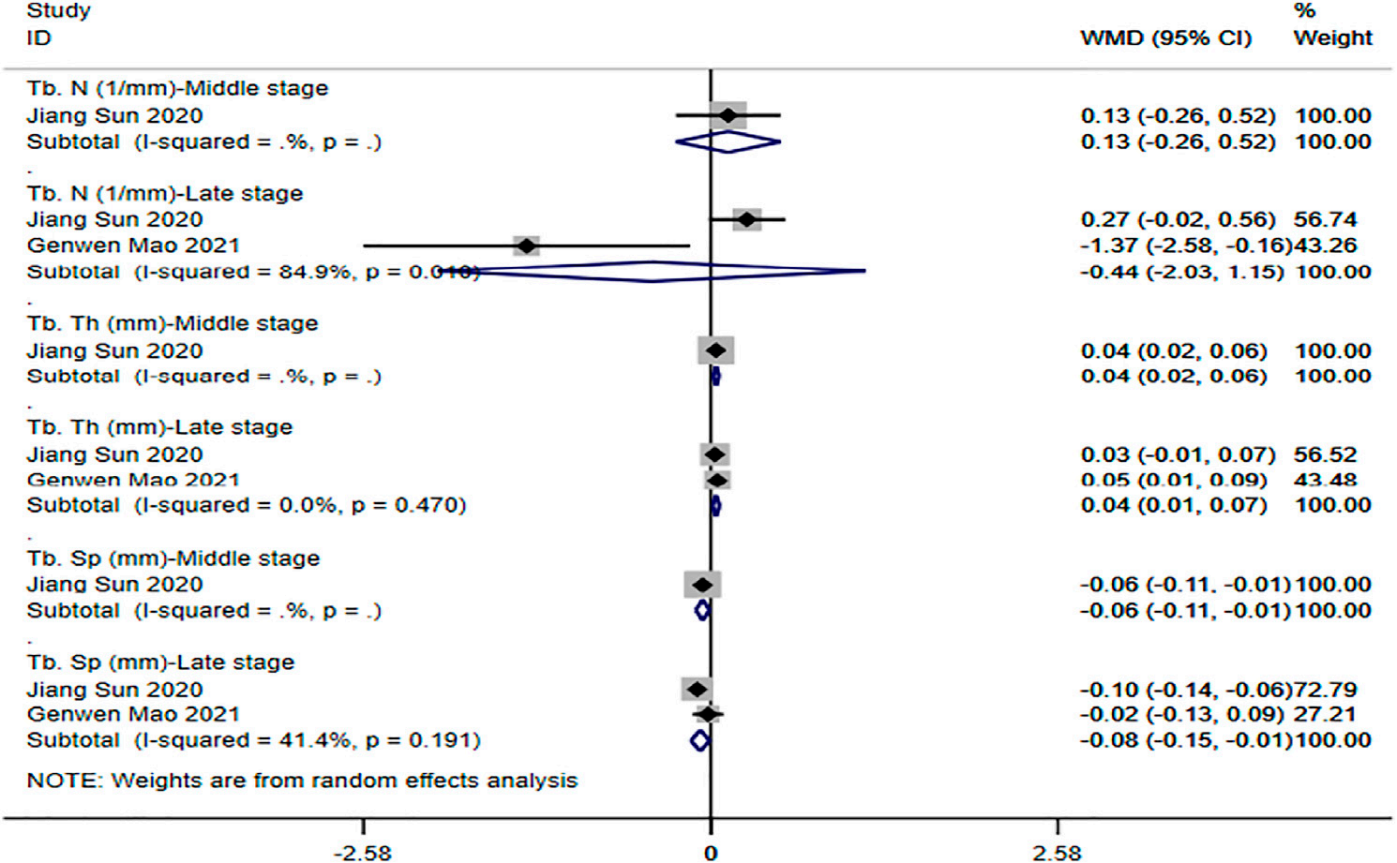
Meta-analysis results of outcome indicators related to new bone formation of magnesium and its alloys vs. titanium and its alloys.

**FIGURE 6:**
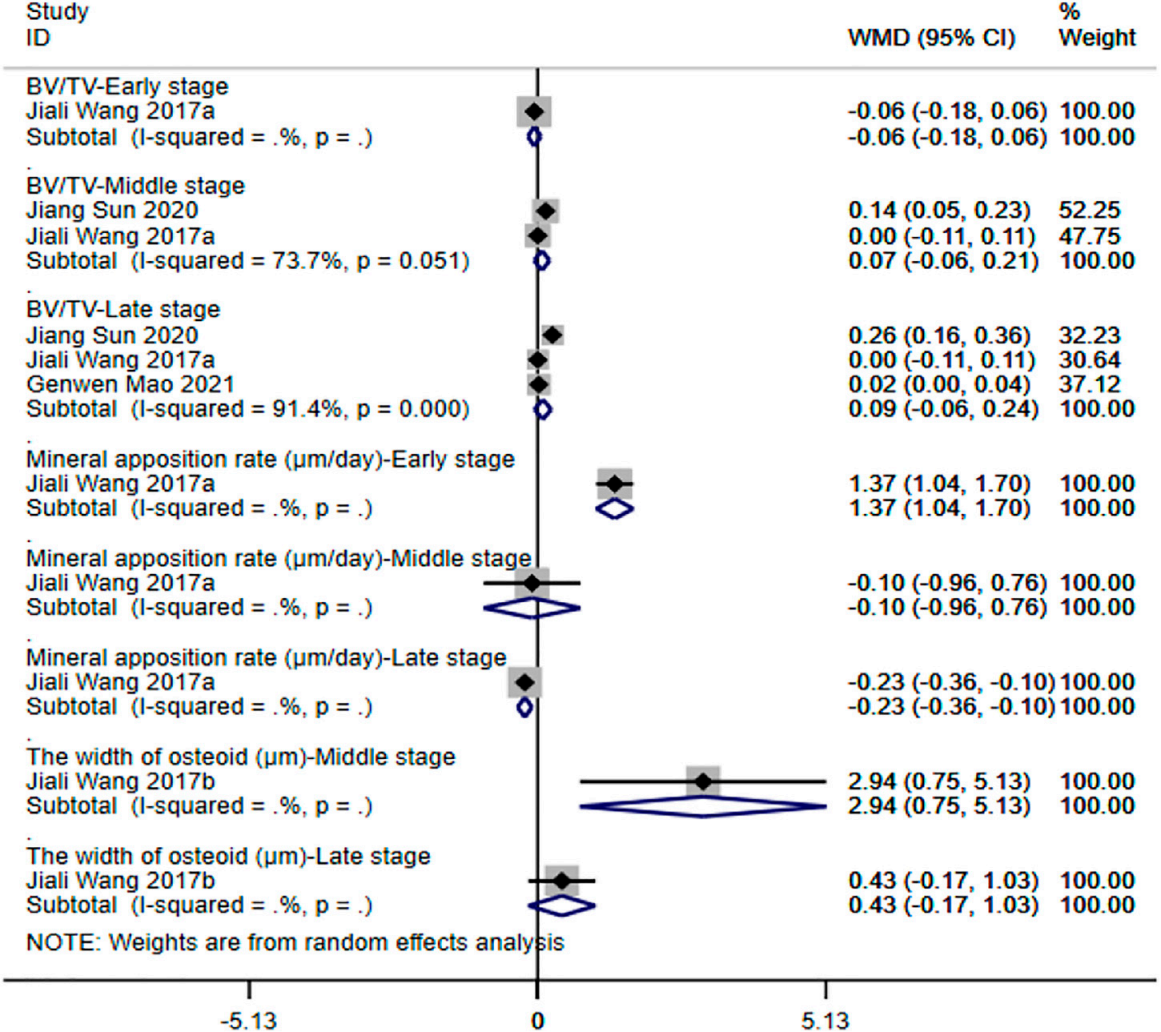
Meta-analysis results of outcome indicators related to new bone formation of magnesium and its alloys vs. titanium and its alloys.

**① Tb. N**: two studies (Mao et al., 2021; Sun et al., 2021) reported the trabecular number of the tendon-bone interface. The results of the random-effect model showed that no significant differences were observed between the two groups with respect to trabecular number (middle stage: WMD = -0.13 [-0.26, 0.52]; late stage: WMD = -0.44 [-2.03, 1.15]). See Figure 5 for further details.
**② Tb. Th**: two studies (Mao et al., 2021; Sun et al., 2021) reported the trabecular thickness of the tendon-bone interface. The results of the random-effect model showed that the trabecular thickness of magnesium and its alloy group was significantly higher than that of titanium and its alloy group, and the difference was statistically significant (middle stage: WMD = 0.04 [0.02, 0.06]; late stage: WMD = 0.04 [0.01, 0.07]). See Figure 5 for further details.
**③ Tb. Sp**: two studies (Mao et al., 2021; Sun et al., 2021) reported the trabecular separation of the tendon-bone interface. The results of the random-effect model showed that the degree of trabecular separation in magnesium and its alloy group was significantly lower than that in titanium and its alloy group, and the difference was statistically significant (middle stage: WMD = -0.06 [-0.11, -0.01]; late stage: WMD = -0.08 [-0.15, -0.01]). See Figure 5 for further details.
**④ BV/TV**: three studies (Wang et al., 2017a; Mao et al., 2021; Sun et al., 2021) reported the ratio of BV/TV. The results of the random-effect model showed that no significant differences were observed between the two groups with respect to BV/TV (early stage: WMD = -0.06 [-0.18, 0.06]; middle stage: WMD = 0.07 [-0.06, 0.21]; late stage: WMD = 0.09 [-0.06, 0.24]). See Figure 6 for further details.
**⑤ Mineral apposition rate**: only one study (Wang et al., 2017a) reported on the indicator. The results showed that the mineral apposition rate of magnesium and its alloy group was significantly higher than that of titanium and its alloy group, and the difference was statistically significant (WMD = 1.37 [1.04, 1.70]) in the early stage. Differences between the two groups were not statistically significant in the middle stage (WMD = -0.10 [-0.96, 0.76]). In the late stage, however, the mineral apposition rate of titanium and its alloy group was significantly higher than that of magnesium and its alloy group (WMD = -0.23 [-0.36, -0.10]). See Figure 6 for further details.
**⑥ The width of osteoid**: only one study (Wang et al., 2017b) reported on the indicator. The results showed that the width of osteoid of magnesium and its alloy group was significantly higher than that of titanium and its alloy group in middle stage (WMD = 2.94 [0.75, 5.13]). No significant differences were observed between the study groups in the late stage (WMD = 0.43 [-0.17, 1.03]). See Figure 6 for further details.

#### Degradation of implants

**FIGURE 7:**
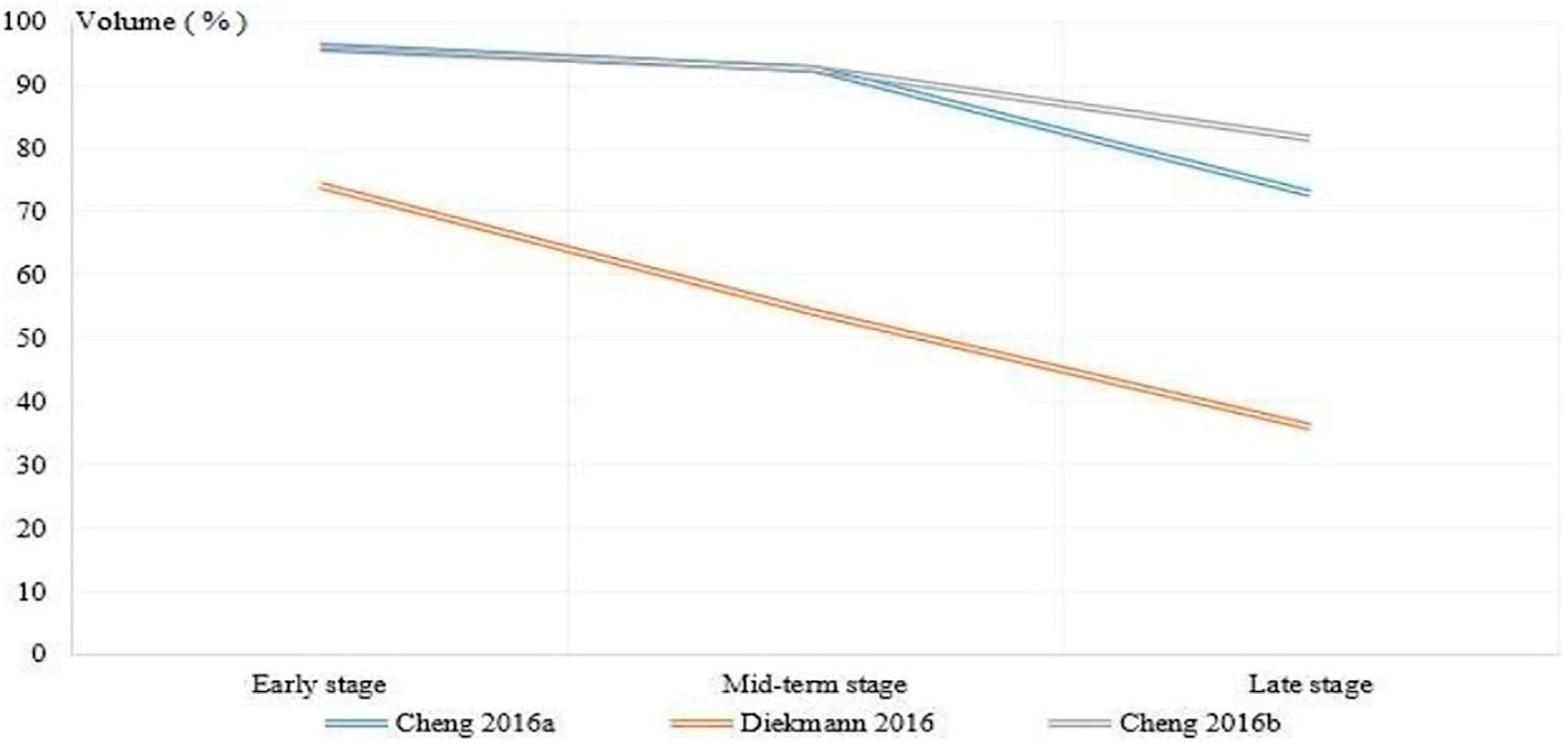
The trend of magnesium and its alloys volume over time.

Four studies (Cheng et al., 2016b; Cheng et al., 2016c; Diekmann et al., 2016; Wang et al., 2017b) reported the degradation of the implant. Since the control group was non-degradable titanium and its alloy, there was no need for comparative analysis. Thus, we only described the trend of magnesium and its alloys with implantation time; see Figure 7 for detail. All results showed that different degrees of degradation of magnesium and its alloys occurred at the end of follow-up. Also, the results of Cheng et al. (2016b), Cheng et al. (2016c), and Diekmann et al. (2016) showed that there were mineral deposits on the screw surface with magnesium screw degradation, and there was a balance between screw degradation and bone-tendon healing. The results of Wang et al. (2017b) showed that the screws were almost completely degraded at follow-up to 16 weeks, and the new bone tissue grew into the bone tunnel.

## Discussion

Magnesium and its alloy had received increasing attention as a potential medical degradable metal, and some products had been used in clinic (Zhao et al., 2016; Li et al., 2020; Wang et al., 2020). However, magnesium and its alloys for orthopedic implants was still in the preclinical stage, especially within the field of ACLR. A large number of original animal studies had been published in the last 5 years, but the results of these studies were inconsistent. Thus, our study was the first systematic review of a comprehensive analysis of seven animal studies meeting the inclusion criteria to explore the specific role of magnesium and its alloys in ACLR, while pointing out problems in current animal studies to reduce the risk of animal study results to clinical transformation.

### Summary of evidence

In our study, meta-analysis was carried out for five studies that reported ultimate load to failure. The results demonstrated that magnesium and its alloys showed the same fixed strength as titanium and its alloys throughout the whole observation period about the FTGTC. In particular, in the late stage, magnesium and its alloys had been gradually degraded but still showed the same fixation effect as titanium and its alloys, indicating that the FTGTC fixed by magnesium and its alloy could fully provide mechanical properties comparable to the FTGTC fixed by titanium and its alloy. The main reason might be that the degradation products of magnesium and its alloy downregulated MMP-13 expression while promoting the expression of BMP-2 and VEGF at the bone-tendon interface, thereby promoting the biological binding of the tendon graft to the surrounding bone tissue (Li et al., 2014; Cheng et al., 2016b). There were also studies showing (Wang et al., 2017a; Wang et al., 2017b) that the degradation products of magnesium and its alloys could enhance the migration, adhesion, and osteogenic differentiation of bone marrow mesenchymal stem cells (BMSCs), thus promoting the mineralization of tendon grafts and achieving the purpose of promoting bone-tendon integration. In addition, compared with the mechanical strength provided by titanium and its alloys, magnesium and its alloys had good biocompatibility, and their elastic modulus and density were closer to human bone, which could provide mechanical properties that were more suitable to human bone. As a result, the incidence of complications such as bone-tendon rupture caused by stress shielding and rejection caused by foreign bodies could be effectively reduced (Staiger et al., 2006; Yazdimamaghani et al., 2017; Al Hegy et al., 2020).

The quantity and quality of new bone formation at the bone-tendon interface were key factors to promote bone-tendon healing and enhanced the biomechanical properties of reconstructed ACL (Lu et al., 2011). The meta-analysis was carried out on four studies that reported outcome indicators related to new bone formation; we found that magnesium and its alloys could be equivalent to titanium and its alloys in promoting increased bone mass (trabecular number and BV/TV) in the middle and late stages of follow-up. Moreover, the trabecular thickness around the bone tunnel in magnesium and its alloy group was significantly better than that in titanium and its alloy group, and the degree of trabecular separation was also significantly lower than that in titanium and its alloy group, indicating that magnesium and its alloys were superior to titanium and its alloys in promoting the formation of new bone. This might be related to the magnesium ions after degradation of the implant significantly stimulated osteogenic differentiation in BMSCs. Unlike clinical patients, researchers struggled to control autonomous activities of animals. Therefore, the earlier the time of new bone formation at and around the bone-tendon interface and the formation of a reliable FTGTC, the earlier it could reduce the risk of failure of the transplanted ACL caused by spontaneous activity in animals. The early, middle, and late stages of new bone formation were not clearly defined in the current field, and meanwhile, according to our division of the research stage, the experimental results included in the research report were their middle and late follow-up research data, which lacked early research data. The possible reason was that there was no unified standard or the specific stage of new bone formation in the current research field cannot be uniformly defined, which led to differences in the description of a new bone formation stage in different studies. Sun et al. (2021) measured the study results at weeks 6, 12, and 16 and analyzed the data at week 6 as early results. According to our definition of the follow-up node, week 6 was in the middle stage and weeks 12 and 16 were in the late stage. In summary, as a weak and critical period of biological binding at the bone-tendon interface (Cheng et al., 2016c), the earlier the new bone formation, the lower the risk of transplanted ACL failure. Therefore, in addition to the measurement results of the middle and late stages, future studies should also pay attention to the measurement of the initial stage results data after ACLR and further explore the timing of the beginning of new bone formation and the time needed to form a solid bone–tendon complex, so as to comprehensively evaluate the effect of magnesium and its alloy in promoting new bone formation throughout ACLR. Also, how to divide the early, middle, and late stages of the experiment scientifically and reasonably is also a problem that needs to be discussed in future research.

Compared with the macroscopic outcome indexes such as trabecular bone and BV/TV which reflect the quantity and quality of new bone formation obtained by imaging examination, the microscopic outcome indexes such as mineral apposition rate and the width of osteoid obtained by histological analysis can further reflect whether the newly formed bone can meet the mechanical properties of the bone-tendon interface (Wang et al., 2017b). Although Wang et al. reported the mineral apposition rate (Wang et al., 2017a) and the width of osteoid (Wang et al., 2017b) in different studies, the results were contradictory in different periods. A possible reason was that different regions were chosen for outcome measures by investigators in magnesium and its alloys and titanium and its alloy group, which might reduce the comparability of the groups and bias outcome data. Thus, contradictory results emerged [Some studies showed that continuity and directionality of collagen fiber and bone mineralization at the outer joint exit were significantly better than at the tunnel openings (Bedi et al., 2009)]. In addition, there was only one study that reported these two outcome indicators, and its results were easily affected by a single study, resulting in a small sample bias (Braun et al., 2013). As a result, future studies need to measure indicators such as mineral apposition rate and the width of osteoid that can reflect the strength of bone-tendon interface integration, so as to further assess whether the strength of bone-tendon integration can meet the requirements of daily activity load. Overall, magnesium and its alloys could promote new bone formation more efficiently than titanium and its alloys, which was essential to reducing stress concentration and gradually transferring the load from the tendon soft tissue to the bone, thereby enhancing the biostrength of the reconstructed ACL (Rodeo et al., 1993). However, there were little data about new bone formation and inconsistent results for the two metrics of mineral apposition rate and the width of osteoid. Therefore, more animal experiments are needed to further explore the ability of magnesium and its alloys to improve the quantity and quality of trabecular bone and promote bone mineral formation at the macro and micro levels by imaging and histology. Meanwhile, it is necessary for future studies to develop uniform measurement standard and select the equivalent observation area as the source of the outcome data to improve the comparability and reliability of the measurement data.

Degradation performance, as a unique advantage of magnesium and its alloys, avoids the limitation of secondary removal of traditional metals and is an important factor that has become the focus of current research. Magnesium and its alloys showed excellent degradation performance in four studies. Although the cavity left after its degradation could be lengthened by nascent bone tissue, it remained unclear whether the incoming bone tissue had sufficient mechanical strength to meet the load at the bone-tendon interface. This was because all the included studies ended the follow-up when magnesium and its alloys were degraded (not yet completely degraded) or the strength of biological binding of the bone-tendon interface could reach the same level as that of titanium and its alloys; the mechanical strength of the bone-tendon was not further explored. Furthermore, although studies by Cheng et al. (2016b), Cheng et al. (2016c), and Diekmann et al. (2016) had shown that the balance could be reached in screw degradation and bone-tendon integration, there was still a lack of appropriate indicators to evaluate the relationship between them, which led to all studies qualitatively describing the relationship between magnesium alloy degradation and bone-tendon integration but lacking objective or quantitative evaluation (Cheng et al., 2016b; Cheng et al., 2016c; Diekmann et al., 2016). In particular, the early stages after ACLR were the critical period to determine the degree of knee functional recovery; if the degradation rate of magnesium and its alloy was too fast, it would lead to premature failure of the implant to provide sufficient mechanical strength, ultimately leading to the failure of ACLR (Cheng et al., 2016c). Therefore, it is very necessary for future studies to comprehensively explore the adaptation rate of the degradation rate of magnesium and its alloy and the healing rate of bone-tendon to finally evaluate its effect in the ACLR process. Additionally, the excessive gas produced during the degradation of magnesium and its alloy led to the formation of air cavity around the bone tunnel, which affected the healing effect, which was also one of the limitations of the current implants. Alloying, surface treatment, and modification might be an important means to overcome the above defects of magnesium and its alloys, and more research would be needed in the future.

In conclusion, compared with the possible “stress shielding ” effect, poor wear resistance, secondary removal, and potential harm of aluminum and vanadium containing titanium and its alloy to the body, not only could magnesium and its alloy provide good mechanical properties, but also its degradation products could also promote the formation of new bone at the bone-tendon interface (Frost, 2004; Costa et al., 2019). However, few original data were available on magnesium and its alloys in improving the quantity and quality of trabecular bone and promoting bone mineral formation. Further preclinical studies need to explore whether the degradation rate is adapted to the rate of bone-tendon integration, whether the gas produced after degradation affects the effect of bone-tendon integration, and whether the degraded newborn bone tissue meets the load of the bone-tendon interface.

### Quality of evidence

Based on a rigorous systematic review, we found that the current evidence quality of magnesium and its alloys in ACLR was low, which reduced the reliability of the experimental results and increased the risk of animal experimental results transforming to clinical practice. Possible reasons included the following.

**1) There was substantial heterogeneity of the included studies.** Although New Zealand White rabbits from male were used as an animal model for ACLR, four bone tunnel diameters, three tendon grafts, three screw specifications, and four follow-up end points were included. In addition, only three studies reported the body weight of animals, and there were differences, which eventually led to greater heterogeneity among studies and reduced the reliability of meta-analysis results. The studies also had great differences in the measures for the same index. For example, the Zwick material testing machine (Cheng et al., 2016b; Cheng et al., 2016c), uniaxial mechanical testing machine (Wang et al., 2017a), and biomechanical testing machine (Mao et al., 2021; Sun et al., 2021) were used to evaluate ultimate load to failure; high resolution-peripheral quantitative computed tomography (Wang et al., 2017b) and Micro-CT (Mao et al., 2021; Sun et al., 2021) were used to measure the data about new bone formation; and Micro-CT (Cheng et al., 2016b; Cheng et al., 2016c) and μCT-scans (Diekmann et al., 2016) were used to measure remaining volume of implants. The difference of measurement methods would affect the consistency and accuracy of experimental results to some extent, thus affecting the reliability of meta-analysis results. Therefore, similar ACLR animal models (including animal species, age, gender, weight, etc.) should be used as much as possible in future studies, and the process of experimental implementation and results measurement should be further standardized to improve the authenticity of experimental results and the reliability of systematic evaluation results.

**2) The normative and unified nature of inclusion of research outcome indicators needs to be further improved.** Although the vast majority of studies had reported the ultimate failure load, which was an indicator of whether magnesium and its alloys could provide good mechanical properties, the focus of other indicators, such as new bone formation, was not consistent, including Tb. N, Tb. Th, Tb. Sp, BV/TV, mineral apposition rate, and the width of osteoid, resulting in a lack of sufficient data for combined analysis of each outcome index. To some extent, it reduced the credibility of the research results. Moreover, degradation, as one of the greatest advantages of magnesium and its alloys, had been concerned by most studies, but these studies qualitatively reported its degradation and only observed whether it is degraded or not. However, the degradation of implants was not associated with new bone formation and gas production in the bone tunnel. To sum up, there was no unified standard or specification indicating which outcome index could reflect the effectiveness and safety of magnesium and its alloys, and there was no comprehensive analysis of the outcome index obtained in the experiment. As a result, the outcome indicators of many current research reports were too different. The inappropriate selection of the outcome index of the experiment might lead to a huge waste of the experiment and the wrong conclusion of the experiment (Ioannidis et al., 2014). Therefore, future research needs to standardize the measurement methods and means of outcome indicators; also, unified standards and norms are adopted to specify indicators that can best reflect the safety and effectiveness of magnesium and its alloys in ACLR, so as to enhance the value of experimental research and avoid the waste of experimental animals.

**3) Insufficient intrinsic authenticity of included studies.** All randomized grouping methods included in the study were unclear, and no hidden grouping was reported. In addition, the unbalanced baseline features of 71.43% (5/7) of the study led to a higher probability of selective bias. Compared with clinical trials, the sample size of most animal experiments was relatively small. Differences in age, weight, gender, and other important baseline characteristics of animals would lead to different responses to the same intervention, thus affecting the consistency and accuracy of experimental results (Hooijmans et al., 2014). Therefore, in addition to the strict implementation of random grouping and hidden grouping in future experiments, the balance of important baseline characteristics was also an important means to reduce the selective bias. Moreover, all included studies did not report blinding of caregivers/researchers or outcome assessors. Although there was no need to blind animals in animal experiments, the researchers in most studies were animal breeders, who might introduce implementation bias and measurement bias due to subjective factors in the process of intervention, result measurement, and evaluation. Therefore, it is necessary to be blind in the intervention implementation and outcome measurement stage to reduce the implementation bias and measurement bias during the experiment and increase the authenticity of the experimental results (Zeng et al., 2013; Tao et al., 2019). Moreover, for the determination of outcome indicators, in addition to the implementation of an effective scientific blind method which can avoid the impact of measurement bias on the measurement results, the qualification of the measurement, and the consistency of the measurement on different animals, the accuracy and scientificity of the validity standards will affect the measurement of the results to different degrees (Sessler and Imrey, 2015). The authenticity of the experimental results can be reduced by the measurement of the experimental results by untrained non-professionals and the lack of uniform standards and norms in the measurement process. The seven studies included in this systematic review did not report on the qualifications of the outcome assessors and the assessment protocols and processes. Therefore, future research should pay more attention to the application of the blind method in experimental design, and at the same time, the specific experimental implementation details should be reported comprehensively, so as to improve the repeatability and reliability of animal experimental results (Dell et al., 2002). Although all the included studies clearly reported all expected results in their methods and results sections, we could not obtain their original research protocols, and we ultimately judged whether they were implemented accordingly and whether all its results were reported in an unbiased manner. Selective reporting of animal experimental research results might lead to publication bias, which might affect the reliability of systematic review conclusions and even lead to opposite conclusions (Korevaar et al., 2011). Government agencies and trade associations should encourage prospective registration of animal studies to obtain raw data (Ritskes-Hoitinga et al., 2014). It is very necessary for future animal studies to share raw data as online appendices (Ritskes-Hoitinga et al., 2014), enhancing research transparency and promoting quality of animal studies.

**4) Insufficient external authenticity of included studies.** External authenticity refers to the extent to which clinical trial results can be reproduced repeatedly in the target population and in the common population (Wu et al., 2011). At present, in the study of the application of magnesium and its alloys in ACLR, rabbits were used as the research object to establish the animal model of ACLR. Although the technique was very mature, the knee joint of the rabbit was in a state of hyperflexion, and the rabbit was jumping rather than walking, so that the model could not accurately replicate the effect of clinical patients’ pressure on the reconstructed ACL (Gulotta et al., 2008). Therefore, the conclusion on the mechanical properties of magnesium and its alloys based on the rabbit ACLR model was difficult to translate into clinical practice. Conversely, the sheep ACLR model had been successfully applied to many preclinical studies involving ACL and showed a mechanism of injury and repair similar to that of humans (Papageorgiou et al., 2001; Madry et al., 2015). Therefore, future studies should expand the variety of animal models of ACLR, using large larger animal models more similar to human ACL damage to more accurately explore the true efficacy of magnesium and its alloys.

### Strengths and limitations of the present study

Key strengths of this systematic review include the following. 1) Based on animal experiments, the real effects and limitations of magnesium and its alloys in ACLR were systematically evaluated and analyzed, and the problems existing in the current field and the direction of improvement were pointed out. 2) Based on the GRADE evidence grading system, the evidence quality of each outcome index was evaluated, and the risk and feasibility of the transformation from animal experimental research to clinical trials were evaluated more scientifically. 3) Based on the internationally recognized risk of the SYRCLE bias assessment tool, we strictly evaluated the intrinsic risk of bias in animal studies and pointed out the problems in the design and implementation of animal studies in the field, while giving suggestions on how to improve the quality of animal study research.

This systematic review has several limitations: 1) The analysis nodes were divided according to the follow-up time, which led to the lack of research data in the early stage of some outcome indicators, and at the same time, some of the research data were not included in the final result analysis. Therefore, future studies need to establish standardized animal models with uniform specifications to explore outcome indicators and measurement nodes that can best reflect the intervention effect. 2) Searching only Chinese and English databases might result in certain language bias. Language bias might decrease the accuracy and universality of systematic review results. 3) Failure to search gray literature and conference abstracts might result in publication bias. Publication bias might be more prevalent in animal experimental studies (Ioannidis, 2012). Therefore, if systematic reviews did not include unpublished studies, they were likely to lead to an overestimation of the effects of the interventions.

## Conclusion

The mechanical properties of the FTGTC fixed with magnesium and its alloys were comparable to those fixed with titanium and its alloys; also, magnesium and its alloys were superior to titanium and its alloys in promoting new bone formation. Although whether the degradation rate of magnesium and its alloy could match the rate of bone-tendon integration and whether the bioconjugation of degraded bone-tendon could meet the exercise load still needs further study, compared with titanium and its alloy, the degradation characteristics of magnesium and its alloy could avoid the injury caused by the second removal of traditional metal, and its degradation products also could promote the formation of new bone, which has good therapeutic potential in the process of ACLR.

However, through a comprehensive analysis of the risk of internal and external bias, quality of evidence, and outcome indicators of included studies, we found that certain limitations were existed in the experimental design, measurement results, statistics, and quality of the evidence. Therefore, future preclinical studies should further standardize the design, implementation, and measurement of animal studies in order to further reduce the risk of the transformation of animal experimental results into clinical practice.

## Data Availability

The original contributions presented in the study are included in the article/Supplementary Material; further inquiries can be directed to the corresponding authors.
