# Supplementary material for: The Role of Biodegradable Magnesium and Its Alloys in Anterior Cruciate Ligament Reconstruction: A Systematic Review and Meta-Analysis Based on Animal Studies
Source: Front Bioeng Biotechnol. 2021 Nov 16;9:789498. doi: 10.3389/fbioe.2021.789498 (PMC8636800; doi:10.3389/fbioe.2021.789498)
Supplement: Supplementary file 1 [file DataSheet1.DOCX]

**Supplemental data:**

**Chinese and English search strategies**

Comments:

In order for non-Chinese readers to understand the Chinese search strategy of this article, we translated the Chinese search terms in the search formula.

**Pubmed**

#1: ("Mg-3Sc-3Y alloy" [Supplementary Concept] OR "WE43 alloy" [Supplementary Concept] OR "Mg-6Zn alloy" [Supplementary Concept] OR "Magnesium"[Mesh]) 219140

#2: biodegradable metal [Title/Abstract] OR degradable metal [Title/Abstract] OR biodegradable alloy [Title/Abstract] OR degradable alloy [Title/Abstract] OR absorbable metal [Title/Abstract] OR magnesium [Title/Abstract] OR Mg [Title/Abstract] 538788

#3: #1 OR #2 598170

#4: "Anterior Cruciate Ligament"[Mesh] OR "Anterior Cruciate Ligament Reconstruction"[Mesh] 14518

#5: anterior cruciate ligament reconstruction [Title/Abstract] OR anterior cruciate ligament [Title/Abstract] OR ACL[Title/Abstract] OR ACLR[Title/Abstract] 25638

#6: #4 OR #5 27351

#7: #3 AND #6 142

**Embase (Ovid)**

#1: 'biodegradable metal':ab,ti OR 'degradable metal':ab,ti OR 'biodegradable alloy':ab,ti OR 'degradable alloy':ab,ti OR 'absorbable metal':ab,ti OR magnesium:ab,ti OR mg:ab,ti 2085174

#2: 'magnesium'/exp OR 'magnesium' 180574

#3: #1 OR #2 2269943

#4: 'anterior cruciate ligament reconstruction':ab,ti OR 'anterior cruciate ligament':ab,ti OR acl:ab,ti OR aclr:ab,ti 32063

#5: 'anterior cruciate ligament reconstruction'/exp OR 'anterior cruciate ligament reconstruction' 14631

#6: 'anterior cruciate ligament'/exp OR 'anterior cruciate ligament' 30479

#7: #4 OR #5 OR #6 36637

#8: #3 AND #7 1075

**Web of science**

(TS=(biodegradable metal OR degradable metal OR biodegradable alloy OR degradable alloy OR absorbable metal OR magnesium OR Mg)) AND (TS=(anterior cruciate ligament reconstruction OR anterior cruciate ligament OR ACL OR ACLR)) 691

**CNKI**

主题：(生物可降解金属 OR 可降解金属 OR 生物可降解合金 OR 可降解合金 OR 镁合金 OR 镁) AND (前交叉韧带重建 OR 前交叉韧带 OR 膝交叉韧带 OR ACL OR ACLR) 77

Subject: (Biodegradable metal OR Degradable metal OR Biodegradable alloy OR Degradable alloy OR Magnesium alloy OR Magnesium) AND (anterior cruciate ligament reconstruction OR anterior cruciate ligament OR cruciate ligaments of knee OR ACL OR ACLR) 77

**Wanfang**

主题：(生物可降解金属 OR 可降解金属 OR 生物可降解合金 OR 可降解合金 OR 镁合金 OR 镁) AND (前交叉韧带重建 OR 前交叉韧带 OR 膝交叉韧带 OR ACL OR ACLR) 86

Subject: (Biodegradable metal OR Degradable metal OR Biodegradable alloy OR Degradable alloy OR Magnesium alloy OR Magnesium) AND (anterior cruciate ligament reconstruction OR anterior cruciate ligament OR cruciate ligaments of knee OR ACL OR ACLR) 86

**3.VIP**

题名或关键词：(生物可降解金属 OR 可降解金属 OR 生物可降解合金 OR 可降解合金 OR 镁合金 OR 镁) AND (前交叉韧带重建 OR 前交叉韧带 OR 膝交叉韧带 OR ACL OR ACLR) 1

Title or keywords: (Biodegradable metal OR Degradable metal OR Biodegradable alloy OR Degradable alloy OR Magnesium alloy OR Magnesium) AND (anterior cruciate ligament reconstruction OR anterior cruciate ligament OR cruciate ligaments of knee OR ACL OR ACLR) 1

**4.CBM**

#1: "生物可降解金属"[常用字段:智能] OR "可降解金属"[常用字段:智能] OR "生物可降解合金"[常用字段:智能] OR "可降解合金"[常用字段:智能] OR "镁合金"[常用字段:智能] OR "镁"[常用字段:智能] 693296

#2: (("镁"[不加权:扩展]) 296605

#3: #1 OR #2 693296

#4: ("前交叉韧带重建"[不加权:扩展]) OR "前交叉韧带"[不加权:扩展] 16473

#5: "前交叉韧带重建"[常用字段:智能] OR "前交叉韧带"[常用字段:智能] OR "膝交叉韧带"[常用字段:智能] OR "ACL"[常用字段:智能] OR "ACLR"[常用字段:智能] 34107

#6: #4 OR #5 34107

#7: #3 AND #6 104

#1: " Biodegradable metal "[common field: smart] OR " Degradable metal "[common field: smart] OR " Biodegradable alloy "[common field: smart] OR " Degradable alloy "[common field: smart] OR " Magnesium alloy "[common field: smart] OR " Magnesium "[common field: smart] 693296

#2: (("Magnesium "[unweighted, extended]) 296605

#3: #1 OR #2 693296

#4: ("anterior cruciate ligament reconstruction "[unweighted, extended]) OR " anterior cruciate ligament "[unweighted, extended] 16473

#5: " anterior cruciate ligament reconstruction "[common field: smart] OR " anterior cruciate ligament "[common field: smart] OR "cruciate ligaments of knee"[common field: smart] OR "ACL"[common field: smart] OR "ACLR"[common field: smart]34107

#6: #4 OR #5 34107

#7: #3 AND #6 104
